# Extracts from the Proceedings of the Odontological Society, London

**Published:** 1862-06

**Authors:** John Tomes

**Affiliations:** F. R. S., President, in the Chair


### Proceedings of Societies.

### EXTRACTS FROM THE PROCEEDINGS OF THE ODONTOLOGICAL SOCIETY, LONDON.

### MONDAY, FEBRUARY 3, 1862.

JOHN TOMES, Esq., F. R. S., President, in the Chair.

Mr. COLEMAN read a paper "On Anæsthesia, considered especially in reference to operatives in Dental Surgery."

After a brief historical sketch of the subject, the author proceeded to consider the effects and uses of chloroform.

The effects of chloroform have usually been divided into four stages, but as there is no sudden transition from one stage to another, the accounts of observers do not all accord.

From my own observation, I should say that the first stages commence with rather agreeable sensations, derived from the first few inhalations, though attended with a feeling of suffocation when too large a quantity of the chloroform is employed; a feeling of warmth is soon felt about the region of the thorax; the intelligence is but slightly impaired and the senses not much altered; the patient sees and hears most of what is going on around him; the pulse is generally accelerated, and respirations are hurried and irregular. He often feels the pulsations of the cerebral arteries like strokes of a hammer upon his cranium. This stage ends, and the second commences with singing in the ears and tingling in the extremities; a mist rises up before the eyes and the patient becomes unconscious; combined with these, there are frequently muscular movements, often excessive and mostly purposeless; the patient talks or shouts, commencing a sentence and losing his ideas before he has had time to complete it. The pulse I have usually noticed to become very feeble towards the close of this stage. In the third stage we have complete unconsciousness; the muscular movements are replaced by rigidity; the eyelids are commonly closed, the pupils contracted, and the eyeballs turned outwards and upwards as in sleep; the pulse usually recovers itself at this stage, and the respirations are quiet and even; stertorous breathing, accompanied with dilation of the pupils, constitute the fourth stage. Beyond this is death, a stage which fortunately I have never witnessed.

The effects on the nervous centers in the different stages are, I believe, somewhat as follows: Towards the close of the first the cerebral lobes are chiefly affected, as are also those nervous centers through which we appreciate pain—whether through suspension of the function of the grey substance of the spinal cord, which is supposed to convey the impressions of pain, or of some nervous center through which it is appreciated, I will not venture to decide. In the next stage the centers of the various senses are impaired, and from the character of the muscular movements, I should apprehend the function of the cerebellum was interfered with. In the third stage the spinal cord first suffers irritation, producing muscular spasm, often resembling tetanic convulsions; but as it proceeds it fails to respond to stimuli, as in the fourth stage, where reflex action ceases, except that of respiration, which continues only so long as the office of the medulla oblongata is preserved, which terminates at the end of the fourth stage.

I would not insist that this order is followed out in every case of anæsthesia produced by chloroform, but I believe it applies to the generality of cases, my conclusions having been drawn from observations on about a thousand administrations that I have witnessed.

From the great diversity of opinion which prevails respecting the action of chloroform on the blood, little appears to be known on the subject. Some contend that chloroform replaces oxygen in the blood, and, by preventing its being arterialised, interferes with the proper nutrition of the nervous centers, and, consequently, with their proper functions; others, that it prevents the carbonic acid being given off at the lungs, producing the same effects upon the nervous centers as the deficiency of oxygen. Many have believed it to be due to carbonic acid formed by decomposition of the anæsthetic; one writer, Dr. Jackson, of Boston, is of the opinion that the chlorine of the chloroform replaces oxygen in the blood. A variety of phenomena lead us, I think, to believe that the first mentioned is probably the true effect of chloroform in the circulation.

Speaking of the dangers of chloroform, the author said:

In considering this part of our subject, let us endeavor to ascertain in what proportion fatal cases occur. Dr. Snow informs us that in his practice he administered chloroform about four thousand times, and met with two deaths, neither of which he considered due to the effects of this agent, and from his accounts of them, one at least certainly appeared to be due to other causes.

Dr. Simpson, in the article "Chloroform," in the last edition of the "Encyclopedia Britannica," though he does not actually give the proportion of fatal cases, says: "Occasional disagreement with, or deleterious influence upon, one in ten or twenty thousand is no sound argument against other patients benefiting from its employment."

Dr. Kidd, who has given great attention to this subject, states that he has witnessed about eleven thousand administrations, and has seen only two deaths. At St. Bartholomew's Hospital, I calculate that chloroform has been administered, in round numbers, about five thousand times, and there have been two fatal cases. In the Crimea, according to M. Flourens, chloroform was given in twenty-five thousand cases without a bad result.

There is little doubt but that many of the deaths attributed to chloroform have resulted from other causes than the deleterious effects of that agent. Shock, loss of blood, and other causes, which, before the introduction of anæsthetics, killed patients on the operating table, should be taken into account, so that, perhaps, one death in ten thousand might not be far from the correct proportion.

How does chloroform kill? is a question as differently answered as most connected with this subject. Dr. Snow was of opinion that it did so by its direct action on the nervous centers of the heart, causing paralysis of that organ. Dr. P. Black, in a pamphlet entitled "Chloroform, how shall we ensure safety in its administration?" expresses his belief that all deaths from it are due to asphyxia, arising from spasmodic closure of the glottis, caused by the irritating effects of the chloroform when not sufficiently diluted with air. He regards it as similar to the mode of death which an animal suffers when immersed in a vessel containing a large proportion of carbonic acid gas, the irritating effects of which cause closure of the glottis, and prevent the escape of carbonic acid from, or entrance of oxygen into the lungs. The same effect occurs in drowning, the water entering the larynx causes closure of the glottis. Dr. Marcet, in a recent paper in the "Medical Times and Gazette," advances the view that this agent, when taken into the circulation in certain doses, will, like alcohol, cause spasmodic closure of the glottis. Some refer the source of death from chloroform, or at least many cases of it, to carrying the administration too far, until the function of the nervous centers controlling the muscles which effect respiration are suspended and the muscles paralysed. Probably animals are thus killed by it, when we see the muscles expanding the ribs first, and the diaphragm next ceasing to act, the heart beating some few seconds after the latter has stopped.

Another mode of death is when the blood is unable to give up its carbonic acid to the air in the lungs, which is too highly charged with vapor of chloroform ; arrest of the blood in the capillaries will take place, and the right cavities of the heart becoming distended with venous blood, it ceases to act. It is probable that deaths have occurred in all the ways here mentioned; but the first noticed—viz., that where the heart's action is arrested through the direct agency of the chloroform upon the nervous centers, is unfortunarely the one least under our control, and does not always appear to be due to any disease in that organ.

The conditions, I believe, most important for the safety of the patient in administering chloroform differ somewhat essentially from those that have been advanced by several high authorities. It is my belief, but I express it with much diffidence, that the more slowly, within certain limits, chloroform is administered, the safer will be the condition of the patient —the view held by the late Dr. Snow, by Dr. P. Black, and by those who consider that death usually occurs through spasm of the glottis. Dr. Simpson, on the other hand, in the article on 'Chloroform,' before referred to, states "the principal error committed in using chloroform in surgery consists in giving it at first in such small doses, or so slowly, as to keep up a state of excitement, instead of inducing a true state of anæsthesia." Dr. Kidd, also, while he deprecates the employment of large quantities of chloroform, appears to disapprove of the plan of inducing anæsthesia slowly, as prolonging the generally admitted dangerous stage of excitement. In this stage the heart's action is usually more excited, and respirations more frequent and irregular, so that a larger quantity of vapor may be taken suddenly into the circulation than in the latter stages, where the action of these two organs is moderate and regular. My own experience, however, does not at all convince me that the stage of excitement is longer or more severe when the chloroform is administered very slowly; on the contrary, I believe there is less excitement altogether than when it is administered rapidly. My friend, Mr. Lloyd, who holds the appointment of administrator of chloroform at St. Bartholomew's Hospital, adopts this plan, and I think with much success.

In addition to the precaution of administering chloroform slowly, I would recommend its not being carried further than is absolutely necessary to produce anæsthesia, which is, I believe, very commonly done. From certain experiments made upon myself, and from watching the effects of chloroform on others, I feel convinced that in the majority of cases, if not in all, the sense of pain is removed before that of touch, and even before the complete absence of consciousness. That different nervous centers are the seats of pain and of the sense of touch has, I think, of late been pretty clearly established.

Besides the usual precautions of making the patient unloose any portions of the dress that might impede the movements of the muscles of respiration—of not administering chloroform too soon after a meal, though the opposite extreme of giving it too long after one is perhaps worse—nothing can be more important than the endeavor to calm a patient's fears before commencing the inhalation. Fear has a most depressing action on the heart and nervous system, and this, added to the effects of the chloroform, has been undoubtedly the cause of many deaths. On this account, I think it is a great advantage to be able to tell your patient that you are not going to send him to sleep, but only to make him unconscious of pain; so that he shall know all that is going on, but not undergo any suffering. In addition to the satisfaction of knowing whether any disease of the heart exists, there is an advantage in listening to that organ, as after doing so, you may, in all probability, be able to assure your patient that he will take chloroform exceedingly well; intelligence that is always received with satisfaction. Patients with valvular disease of the heart appear to take chloroform very satisfactorily, although the existence of this, or disease of the lungs, should, if possible, make us more than usually cautious when administering it.

In employing chloroform in dental operations, the first question that may be asked is, "Are operations on the teeth sufficiently painful to justify the employment of a means by which the life of a patient may be hazarded?" In answering this question, we naturally ask another, viz.,—What proportion of risk does a patient run who takes chloroform? We came to the conclusion, when considering the proportion of fatal cases of chloroform, that they were about one in ten thousand. As every dentist probably removes more than ten thousand teeth during the whole of his practice, if he employed chloroform in every operation, he might expect one fatal case to occur to him in the course of his life-time, an event which no sensible man would desire.

I think it would be a safe rule to lay down, never to recommend chloroform for the extraction of one or even two teeth, provided there appeared nothing remarkably difficult about their removal; but not to refuse it if the patient very much requested it, and then to apply it only in the small quantity before recommended. If only carried thus far, I do not believe the proportion of fatal cases would exceed one in fifty to one in a hundred thousand. In looking over the recorded cases of those who have died after taking only a few inhalations, if we exclude those where the patient was evidently asphyxiated from an over-dose of the agent, I do not think they will be found to exceed ten in number.

When, however, our patient requires four or more teeth removing at one time, our course then becomes clear, and on the ground of humanity, unless an objection is expressed, we may justly urge its employment.

The author described his instrument for holding the mouth open, and an inhaler adapted for administering chloroform by means of the nostrils, in operations about the mouth, and proceeded to say:

Before offering some remarks on local anæsthesia, it is perhaps right to say a few words upon the best means to be adopted should any alarming symptoms occur while operating with chloroform. Ordinary syncope is not uncommon, especially when much blood is lost. In such cases the horizontal position, or what is still more effective, pressing the head down upon the knees, would, in all probability, suffice to restore the patient; but we must remember that the blood is charged with a very depressing agent, so we must excite as soon as possible the full action of the muscles of respiration, to get rid of it at the lungs, by dashing cold water on the face and chest, and moderately stimulating the mucous membrane of the nostrils, etc. It may be also advisable to administer stimulants; this should be done through the rectum, for fear of any getting into the trachea if administered in the ordinary way. I have witnessed many cases of this accident, but have had only one case occurring in my own practice. My patient was a lady of about twenty-seven years of age, for whom it was necessary to remove every tooth she possessed. The gentleman who administered the chloroform was her relative—a physician of some eminence. She came to me about twelve o'clock in the day, having eaten very little breakfast. She was pale, apparently much alarmed, and her pulse was quick and feeble, on which account a little wine was given her. She took the chloroform well, which had to be applied two or three times, as she came round very rapidly. When all her teeth had been removed but six, her face suddenly became pale, and her lips rather livid, her pulse became very feeble, and soon could not be fel. I applied a stethoscope over the heart, but could not hear it acting. The recumbent position was immediately adopted, fresh air admitted by the window, and cold water dashed on to the face and chest. This soon produced one or two sighing respirations, followed by return of the pulse and subsequent animation. On the same day the lady dined with her relative, and, under his advice, returned to me in the afternoon, when the six remaining teeth were removed under chloroform, which she took remarkably well.

Should the means proposed—viz., the horizontal position, the admission of fresh air, the application of cold water, and introduction of brandy into the rectum, fail to restore animation, recourse must be had to artificial respiration. Should the accident occur when only one medical man is present, Marshall Hall's method is that most easily performed; but when there is plenty of assistance, I should prefer the plan of placing the patient in the supine position, pressing upon the ribs and abdomen, and remove the pressure about twenty times in the minute. It is an advantage to be able to watch the patient's countenance during this operation, and to see that air freely enters and is expelled from the lungs. Should it not do so, tracheotymy should be performed, as Dr. Marcet has recently shown that the chloroform in the system may produce spasmodic closure of the glottis. Of course, before doing so, the tongue should be drawn forward, as this might, by falling back, have closed the glottis. I would myself keep up artificial respiration for an hour before giving up a case as hopeless. Galvanism has been found of little avail in these cases, and probably, from the time lost in employing it, worse than useless. If the veins of the neck and face appeared much distended, an external jugular vein might be opened, but artificial respiration will usually soon relieve the right cavities of the heart.

The author having made some remarks upon local anæsthesia by means of cold, electricity, or other agents, concluded by saying:

I do not deny that certain bodies, as aconite, etc., act locally upon the peripheral extremities of nerves; but I think Dr. Simpson is not far wrong in the statement, "that in the human subject the degree of partial or superficial anæsthesia which is capable of being produced by chloroform in the state of liquid or vapor, is never sufficiently great to allow of the part being cut or operated upon without pain, at least not in man, probably in the lower animals."

Mr. Rogers asked whether there were any symptoms by which the operator can determine when the patient is in that state when there is no sensibility to pain, though the patient shrinks from the touch. It would be of great importance, of course, to be able to decide that point.

Mr. Coleman did not think there was any test by which to tell exactly when that state had arrived.

Mr. Cattlin wished to elicit the opinion of the Society as to whether chloroform is a warrantable remedy in Dental Surgery, and under what conditions its use may be desirable. It has unfortunately fallen to my lot to come to the conclusion, after considerable experience in the matter, that there is no amount of experience which can guide us in any way as to the application of chloroform to dental purposes. I do not think it is a matter as to whether there is disease of the lungs or disease of the heart, or anything of the kind. I believe there is some peculiar idiosyncrasy about some patients which renders them more liable to be poisoned by chloroform than others, in the same way that one person is affected by opium in a poisonous manner and another in a medicinal manner. There are several cases to which I would allude, which show plainly that there must be some peculiar idiosyncrasy on the part of patients in that respect. I remember one—that of a lady whom I attended. I had exhausted all my arguments in endeavoring to persuade her not to take chloroform; but as she went so far as to say that she would go to Scotland—to Glasgow—to have it administered if I refused, I put twenty minims of chloroform in one of Snow's inhalers; and she had scarcely taken twenty inhalations before—I must tell you she was dressed for an evening party—I noticed the whole of her arms and chest becoming mottled, and she fell back insensible in the chair, and was, in fact, in a dying condition—no pulsation of the heart being perceptible. The window was opened and everything done to restore animation, but she was in a state approaching death certainly for at least from fifteen to twenty minutes. I think that is the all-important subject, for the large number of persons whose health has been injured by the application of chloroform for dental purposes is greater than we have any idea of. I remember two cases of dispensary patients—persons of low health, and very likely low food, probably living in ill-ventilated houses—where, as the immediate consequence of the administration of chloroform, sore throat of a sloughing character made its appearance, which I can attribute to no other cause than the depressing influence of chloroform upon the system. Prior to the sloughing throat, they felt extremely weak—scarcely able to walk—and then the sloughing sore throat followed. I can call to mind some instances, in fact several, in which for at least six months after the ordinary application of chloroform, persons have lost their memory—they have not had the same amount of memory as before the application of the vapor. I also recollect the case of a person having been subject to syncope, never having been subject to it prior to the application. I have also had reported to me one or two cases of insanity following the administration of chloroform. Certainly, in one of those instances there was a very strong hereditary disposition to insanity, but it occurred only one day after the chloroform had been administered. There have been some very useful and excellent remarks in Mr. Coleman's paper, one being the effect of fear upon the patient. I believe fear to be most injurious, and that we should not administer chloroform where it exists to any extent. Mr. Coleman has omitted to mention two precautions which are necessary prior to the administration of chloroform, which are, first, that the patient should not have taken food for several hours (which is very important), and secondly, a plan which I invariably adopt—the administration of a stimulant—a glass or two of wine, or some brandy and water. This, of course, is very much regulated by the state of the pulse at the time. I believe that the administration of stimulants would prevent very much of the faintness and depression which we often get from chloroform. I did not hear very well here, but, as far as I understood Mr. Coleman, he said that the most useful application of chloroform has been in minor cases. I certainly have found advantage in applying half a drachm, we will say, on a piece of sponge or cotton-wool, and rubbing the gums and palate with it. I do not pretend to say that it has any particular local effect; but I think you then just get so much of the chloroform mixed with a large quantity of atmospheric air (for that is an important point—the great safety in the administration of chloroform is to administer it very largely diluted with air) as will, to a great extent, render the operation painless. I have never met with any bad symptom when it has been administered in that way.

Upon the motion of Mr. Sercombe, the discussion was adjourned till the meeting after next. A vote of thanks was accorded to Mr. Coleman for his paper, and the proceedings then terminated.—*Dental Review, London.*

